# Speciation and genetic diversity in *Centaurea* subsect. *Phalolepis* in Anatolia

**DOI:** 10.1038/srep37818

**Published:** 2016-11-25

**Authors:** Jordi López-Pujol, Sara López-Vinyallonga, Alfonso Susanna, Kuddisi Ertuğrul, Tuna Uysal, Osman Tugay, Arbi Guetat, Núria Garcia-Jacas

**Affiliations:** 1 Botanic Institute of Barcelona (IBB-CSIC-ICUB), Pg. del Migdia, s/n, ES-08038 Barcelona, Spain; 2 Faculty of Science and Art, Selcuk University, TR-42031 Konya, Turkey; 3 Department of Biology, College of Sciences, Northern Border University, Arar, Saudi Arabia

## Abstract

Mountains of Anatolia are one of the main Mediterranean biodiversity hotspots and their richness in endemic species amounts for 30% of the flora. Two main factors may account for this high diversity: the complex orography and its role as refugia during past glaciations. We have investigated seven narrow endemics of *Centaurea* subsection *Phalolepis* from Anatolia by means of microsatellites and ecological niche modelling (ENM), in order to analyse genetic polymorphisms and getting insights into their speciation. Despite being narrow endemics, all the studied species show moderate to high SSR genetic diversity. Populations are genetically isolated, but exchange of genes probably occurred at glacial maxima (likely through the Anatolian mountain arches as suggested by the ENM). The lack of correlation between genetic clusters and (morpho) species is interpreted as a result of allopatric diversification on the basis of a shared gene pool. As suggested in a former study in Greece, post-glacial isolation in mountains would be the main driver of diversification in these plants; mountains of Anatolia would have acted as plant refugia, allowing the maintenance of high genetic diversity. Ancient gene flow between taxa that became sympatric during glaciations may also have contributed to the high levels of genetic diversity.

The Mediterranean Basin is considered one of the most important hotspots of the world^1^ because of its high concentration of endemic species. Up to 60% of these are specifically narrow endemics^2^. Southern Turkey stands out especially and shows a wealth of endemic species^3^. According to some authors^4^, percentage of endemism reaches 30% of the flora with a maximum concentration in the areas of transition where the floras of different regions meet. Anatolia constitutes a crossroads of three floristic domains: Pontic, Irano-Turanian and Mediterranean^5^. The Mediterranean floristic domain is confined to the western and southern coastal strips and adjacent mountain ranges, mainly Western and Central Taurus, with some minor enclaves on the coast of the Black Sea^3^. It is worth noting the definition of a diagonal line, known as the Anatolian Diagonal (Fig. 1), formed by a long mountain range with heights of 3000–4000 m, which has served as a dispersal corridor for a number of plants and animals from northeast Anatolia and the Black Sea region to south Anatolia^3^. Mountain ranges have acted as refugia for Mediterranean species during glaciations^6^, being the main specific refugia the Taurus, the whole W Anatolia and the Amanus.

In this context of high endemicity, one of the most diverse and most characteristic genera of the Mediterranean flora^5^ is *Centaurea* (Compositae-Cardueae) with over 250 species^7^. *Centaurea* constitutes an excellent model for analyzing the speciation and diversification processes in the region. The main centres of diversification of *Centaurea* coincide largely with the main refugia defined in the Mediterranean^7^,^8^,^9^, one of them being the Mediterranean part of the Anatolian Peninsula^6^. In fact, *Centaurea* is the third genus with more endemic species of Turkey^10^ and the number of species is constantly increasing since the publication of Davis *Flora of Turkey*^11^. Latest estimations suggest that there are 159 species in the area, 118 of them endemics^12^. *Centaurea* is also notable for the frequent introgression and hybridisation^9^ and references therein, which in some cases has offered a window into the history of the species by revealing gene flow between species not sympatric at present^13^.

Amidst the different sections of genus *Centaurea* that show specific diversification in the Mediterranean hotspots, we will focus on sect. *Centaurea* subsect. *Phalolepis*. All the nine Anatolian species of subsect. *Phalolepis* listed in the *Flora of Turkey* are narrow endemics^11^. Two of them, *C. hieropolitana* Boiss. and *C. tossiensis* Freyn & Sint. ex Freyn, fall outside *Phalolepis* according to the results of the latest molecular survey of *Centaurea* and should be included in a new section, sect. *Hierapolitanae*^9^. The species that remain in *Phalolepis* are *C. amaena* Boiss. & Balansa, *C. antalyensis* H. Duman & A. Duran, *C. aphrodisea* Boiss., *C. cadmea* Boiss., *C. dursunbeyensis* Uysal & Köse, *C. luschaniana* Heimerl ex Stapf, *C. lycaonica* Boiss. & Heldr., *C. lycia* Boiss., and *C. wagenitzii* Hub.-Mor.

On morphological grounds, Turkish species of *Phalolepis* share a very similar morphology, which was reviewed in depth recently^14^. In sharp contrast with Greece where most of species were at some point subordinated to other widespread taxa^15^, species of *Phalolepis* described from Turkey are fairly undisputed. They are usually mountain chasmophites on limestone, with the exception of *C. wagenitzii*, which grows on serpentines at sea level.

We chose the group of *Phalolepis* species as a subject for phylogeographic studies for three reasons: firstly, the group is morphologically cohesive and its limits have been recently reviewed and redefined^9^; secondly, there are no doubts on the taxonomy of the species, thanks to a recent morphological review^14^; and thirdly, subsection *Phalolepis* has been subject of a recent study on speciation in Greece^15^, and a similar study in Turkey would allow a comparison of results of high interest. The study in Greece concluded that it was a case of allopatric speciation by area fragmentation mediated by the special topography of the mountains of Greece, which favored isolation, and pointed out the character of refugium of the region during the Pleistocene glaciations^15^.

As in the case of Greece, we selected microsatellites, a highly variable type of marker, for analyzing subsect. *Phalolepis* in Turkey. Microsatellites have the advantages of codominant inheritance, reproducibility and high levels of variability, and are extensively used for analysing genetic structure. Previous studies using microsatellites have resolved the population structure of other species of *Centaurea*^16^,^17^,^18^,^19^,^20^ and have unraveled the speciation process in *Phalolepis* in the refuge of continental Greece^15^. Besides population analyses using microsatellites, ecological niche modelling (ENM) has also been employed to get insights into the paleodistribution of species. ENM represents an independent method of biogeographic inference on a given species, thus being an ideal complement to the classical genetic methods^21^.

Our specific objetives are the following: (1) to investigate population structure by estimating genetic diversity within and between narrow endemics using microsatellites, and compare the results with those in Greece for verifying whether the evolution of the endemicity in *Phalolepis* follow a similar pattern; (2) to analyse the correlation of morphological species and genetic clusters; (3) to explore the levels of ancient and recent gene flow and verify the extent of introgression; (4) to verify whether the range expansions/contractions of the studied *Centaurea* endemics is related to the special physiography of Anatolia using ecological niche modelling in *Centaurea cadmea*, the only species in this study that shows enough occurrences as to build a model.

## Results

### Genetic diversity

All surveyed loci were polymorphic for all the populations (except *21D9* for LY, LU, LYC1, AMA, and CA3). We detected a total of 97 alleles, ranging between four (locus *21D9*) and 29 (locus *12B1*); average was 13.9 alleles per locus. At population level, the mean number of alleles per locus (*A*) ranged between 4.286 in populations LYC1, LYC2, WA2, and CA2 and 7.286 in population LU (Table 1). We detected exclusive alleles for many populations, even though numbers were low (from one to five; Table 1) and frequencies were low too (mean = 0.065). Population LYC2 showed the lowest value of expected heterozygosity (*H*_e_ = 0.491), whereas population WA1 harboured the highest value (*H*_e_ = 0.669; Table 1). The least variable species was *C. lycaonica* (*H*_e_ = 0.498), whereas the highest genetic diversity was shown by *C. antalyensis* (*H*_e_ = 0.655; Table 1).

Only one loci pair showed evidence of linkage disequilibrium (*28A7* vs. *17E3*). Many individual loci (42 out of 77 possible cases) exhibited significant deviations from H–W equilibrium expectations (*P* < 0.05); accordingly, *F*_IS_ values at population and species level were positive and significantly different from zero (Table 1), which can be attributed to inbreeding and/or to the occurrence of null alleles^22^. Given that the frequency of null alelles in our dataset is very low [range = 0.0003 (*21D9* locus) − 0.178 (*12B1* locus); average = 0.095], we do not expect significant biases in our genetic analyses (it has been suggested that biases are negligible when null alleles are present at frequencies below 0.200^22^). However, there are positive correlations between their frequency and *F*_IS_ values within (*N* = 77, *R*^2^ = 0.760, *P* = 0.000) and across populations (*N* = 7, *R*^2^ = 0.573, *P* = 0.049), indicating that the significant deviations from H–W equilibrium can be partly attributed to the presence of null alleles.

All but one genetic divergence values between populations based on *F*_ST_ were significant (*P* < 0.05). These values ranged from 0.027 (LU-LYC1) to 0.297 (LYC2-AMA), with a mean of 0.198 (Table 2). The *F*_ST_ values between pairs of species were significant in all the cases, and somewhat smaller than the *F*_ST_ values between populations (Table 3): they ranged from 0.057 (*C. lycaonica*-*C. luschaniana*) to 0.217 (*C. lycia*-*C. amaena*), with a mean of 0.135. Corrected *F*_ST_ values for null alleles showed very similar to those found using the uncorrected dataset (the differences were only ca. 5–10%), both between populations and between species (Supplementary Tables S1 and S2); the effects of null alleles on the genetic structure of populations, thus, would be negligible.

According to Evanno’s approach, *K* = 10 was the most likely number of genetic clusters for Structure simulations, as this is the only clear peak. The ln Pr(*X*|*K*) also reached a plateau when *K* = 10 (Supplementary Fig. S1). Although several of the studied populations had their “own” clusters, other populations (LY, LU, LYC1, and WA1) showed a high degree of admixture (Fig. 2). Notably, not all the grouping schemes input in AMOVA were significant. The within-population component accounted for most of the total variation (84.42%), the among-population component contributed much less (15.53%), whereas the among-taxa component (i.e., when the populations were grouped into species) was negligible (0.05%) (Table 4). Even though the percentage of variance explained by the first two components was not very high (52.90%), the PCoA analysis at population level was in close agreement with both the Structure and (particularly) with AMOVA results: populations are relatively isolated among them, with no signs of taxonomically meaningful aggrupation (i.e. all populations belonging to a same species were invariably located in different quadrants; Fig. 3).

The first two assigned barriers in the Barrier analysis (Supplementary Fig. S2), mainly separated the populations located on western Taurus Mountains (that is, those around the Gulf of Antalya). With the third barrier (*B* = 3), separations in other regions (Pontic Mountains, central Taurus Mountains) started to be evident, although some separations (LU vs. LYC1, LY vs. CA2, and WA1 vs. WA2) did not appear until *B* = 8. The BayesAss analysis confirmed the absence of recent gene flow between populations: all but one pairwise *m* values were at least one order of magnitude below 0.114 (this figure separates real migration from noise; Table 5). Regarding historical gene flow, as estimated with the software MIGRATE-N, *Nm* values were relatively low (from 0.263 to 1.193, averaging 0.466; Table 6). Total immigration rates were highest for 1LYC, 1WA, and 2WA (all with *Nm* > 5), whereas the populations that showed total emigration rates > 5 were LU, 2WA, and 2CA (Table 6).

### Ecological niche modelling

According to the MaxEnt jackknife tests of variable importance, the precipitation variables were more informative for the model than the temperature ones (the more informative variables were, by far, precipitation of the driest month and precipitation seasonality; Supplementary Fig. S3). The present-day distributional predictions for *C. cadmea* were largely congruent with the known species occurrences, although other areas appear as suitable (shaded in Fig. 4), such as mountains of NW Anatolia and some scattered areas along the western and central Taurus Mountains (Fig. 4A). Projections of the species niche to the LGM climate produced considerably different maps of presence/absence. With the CCSM model almost all the Anatolian Peninsula appears as suitable, leaving only as unsuitable the eastern and southern coastal areas (Fig. 4B). The MIROC model showed that most of the mountainous areas of the Anatolian Peninsula were suitable for *C. cadmea* at the LGM with the exception of the plateau region of central Anatolia (Fig. 4C). Both LGM projections show a considerable increase of the potential area of *C. cadmea* compared to the present time, even for the likely more “realistic” MIROC model (with an increase of about four-fold of the suitable area, compared to an increase of ten-fold for the CCSM). The LGM projections should be, however, treated with extreme caution because the uncertainty of projecting the present-day ENM to the LGM should be added to that resulting from the use of a very small number of occurrences.

## Discussion

### Genetic diversity

Genetic theory predicts low levels of genetic diversity for narrow endemic species because of a combination of traits usually associated with rarity: low population sizes, isolation of populations, inbreeding, and ecological specialization^23^,^24^. In support of this view, several meta-analyses have demonstrated that there is an association between rarity and low genetic variability^25^,^26^,^27^; such association is even more compelling for those species that are limited to one or very few localities, with dozens or, at most, a few hundreds of individuals (often referred as “extremely narrow endemics”, ENEs^28^). In the Mediterranean, two biological traits are often present in the endemic flora: production of few and small flowers, and low investment in pollen and seed production^29^. Both traits might also have a role in the association between rarity and low genetic diversity. Accordingly, examples of Mediterranean narrow endemics showing very low levels of genetic diversity are abundant, e.g. *Anchusa crispa*^30^, *Aquilegia barbaricina/A. nugorensis/A. nuragica*^31^, *Coristospermum huteri*^28^, or *Zelkova sicula*^32^.

Contrarily to the expected patterns for narrow endemics, Anatolian species of subsect. *Phalolepis* are not genetically depauperated at all (mean *A* = 5.643; mean *H*_e_ = 0.580; Table 1). Even the most restricted taxa such as *C. antalyensis, C. cadmea* subsp. *cadmea*, and *C. lycaonica* (all are classified as CR, see below in Material and Methods) have moderate to high levels of genetic diversity, and *C. antalyensis*, only known from the type locality and having less than 250 individuals, harbors the highest levels of variability in terms of expected heterozygosity (*H*_e_ = 0.655; Table 1) among all studied taxa. Genetic diversity values in Turkish species of subsect. *Phalolepis* are among the highest within the genus (mean *A* = 5.160; range 3.100–7.286; mean *H*_e_ = 0.504; range 0.073–0.779; Supplementary Table S3), and are comparable to the values obtained for a series of narrow endemics of the same subsection from the mountains of Greece using the same set of microsatellites (*A* = 5.363; *H*_e_ = 0.587)^15^. Comparison with the “reference” values for plants (*H*_e_ = 0.420 for endemic species, *H*_e_ = 0.620 for widespread ones) provided in the meta-analysis of Nybom^27^ is another proof that our study species conserve considerable microsatellite genetic variability in spite of their rarity.

Our results, as well as the growing number of studies reporting narrow endemic species with unexpectedly high levels of genetic polymorphism^33^,^34^ suggest that the geographic range is not always a good predictor of genetic diversity in plant species. It is generally agreed that a complex network of factors shape genetic diversity in plant populations, which are usually classified into two categories^35^: (1) intrinsic biological properties of the species (i.e., life-history traits and ecological interactions) and (2) extrinsic dynamic processes which affect species (i.e., historical factors that may include occurrence of bottlenecks, divergence events, or Quaternary expansions/retreats). Species of *Centaurea* share most of their biological traits: they are usually protandrous and usually self-incompatible, and they have entomophilous pollination by a wide range of insects^36^,^37^,^38^. Thereafter, intrinsic factors should be discarded and differences in polymorphism between the studied *Centaurea* species (Table S3) are more likely extrinsic and attributable to their evolutionary history.

The endurance of glacial-interglacial cycles in mountain glacial refugia in the Pindus Mountains of Greece was the main reason provided for the high levels of polymorphism detected in the Greek taxa of subsect. *Phalolepis*^15^. Likewise, the relative environmental stability of another mountainous refugium in the Pre-Pyrenean Mountains has been suggested as an explanatory factor for the moderate microsatellite diversity in *C. emigrantis* and *C. tripontina*^39^. The Pindus and the Pre-Pyrenean regions range among the “phylogeographic” glacial refugia recognized within the Mediterranean Basin^6^. The southern Anatolian Peninsula and especially the western and central sections of Taurus Mountains is home of several refugia^6^, and is also characterized by a high concentration of plant diversity and endemism^4^.

Several factors have contributed to the refugial character of the Taurus: the rugged topography of these mountains with peaks surpassing 3000 m, its closeness to the sea, which would have constituted a continued source of moisture even at the most arid phases of the Pleistocene; and, in contrast to the Pyrenees or the Alps, the lack of major ice-sheets. Only the peaks over 1900 m were glaciated^40^. This scenario is consistent with the most recent paleoecological reconstructions for the Taurus Mountains based on pollen sites or climatic simulations: the mountains would have been covered by temperate/boreal forests and parklands instead of more arid formations such as desert or steppe^41^,^42^,^43^.

The results in the montane species of *Centaurea* studied by our team^15^,^39^ clearly support this scenario. *Centaurea* species, like other mountain endemics, would have found abundant favorable pockets throughout the unglaciated, relatively moist Mediterranean mountains, and survived the repeated glacial/interglacial cycles by altitudinal migrations without large geographical displacements. These movements would have favored secondary contacts at the glacials and populations would have admixed, largely blurring the genetic differentiation produced by periods of isolation at the interglacials. This process was favored by the much longer duration of glacials compared to interglacials and has also been proposed for other plants from the Mediterranean mountains^13^,^34^,^44^. Genetic connectivity is also present in Anatolian species of subsect. *Phalolepis* (*F*_ST_ = 0.198) and the results of ENM constitute another indirect evidence because the suitable habitat projected at the LGM is significantly increased for *C. cadmea* compared to the present time (Fig. 4). The increase of LGM suitable areas may be common to the other studied Turkish species of subsect. *Phalolepis*, given that almost all of them have the same ecological requirements, and contacts between populations would have been frequent.

### Systematic and evolutionary implications

The results of the genetic study only partially coincide with the morphological classification usually accepted. The Structure analysis defines individual clusters for *C. amaena* and *C. antalyensis*, and partly for *C. wagenitzii* (Fig. 2). By contrast, several other species share a common gene pool (cases of *C. lycaonica* and *C. luschaniana*, along with a population of *C. lycia*). Besides, *C. cadmea* cannot be defined as a species because each population forms a different gene cluster (Fig. 2), which is very evident in the PCoA (each population falls into a different quadrant; Fig. 3). It is likely that *C. cadmea* is in active process of speciation and in fact there is a morphological reason to support this hypothesis. The northern populations have been segregated as a different subspecies, *C. cadmea* subsp. *pontica* Wagenitz ex Y.B. Köse & Ocak. This case is similar to Greek *C. chrysocephala* Phitos & Georgiadis, a species that could be in active speciation process in its most isolated population in Meteora Mountains^15^.

The results of the AMOVA analysis also indicate that the species cannot be defined as independent entities; the among-taxa component is notoriously low (0.05%; Table 4). This difficulty often arises when dealing with taxa that are scarcely divergent genetically^45^, as in the present case (Table 3). The use of the ITS region and plastid markers in *Centaurea* has usually resulted in a general lack of resolution: the resulting phylogenies consist usually of large polytomies^9^. Introgression and incomplete lineage sorting following recent speciation are usually invoked as the major causes of species-level polyphyly^46^. Speciation of *Centaurea* subsect. *Phalolepis* in Anatolia is recent because an age of 1.55 (0.32–3.13) Ma was assigned to the Turkey/Cyprus haplotype^9^. We will discuss in terms of current and former gene flow the reasons that make us think that cases of shared gene pool are due to introgression in a recently differentiated group.

**Figure 1:**
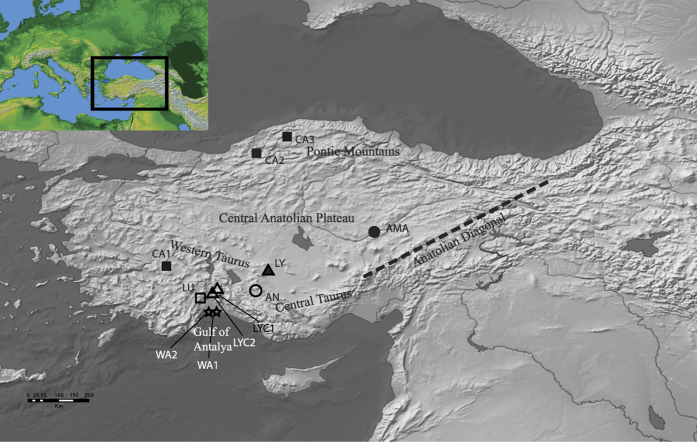
Location of the sampled populations for the seven species studied. Symbols for the sampled populations: • = *C. amaena*; ○ = *C. antalyensis*; ■ = *C. cadmea*; □ = *C. luschaniana*; Δ = *C. lycia*; ▲ = *C. lycaonica*; ☆ = *C. wagenitzii*. This figure has been generated with ArcGIS v. 9.3 (ESRI, Redlands, CA, USA) and modified using Adobe Illustrator CS5.1 (Adobe Systems Incorporated, San Jose, CA, USA). Map layers were obtained from the site www.naturalearthdata.com.

**Figure 2:**
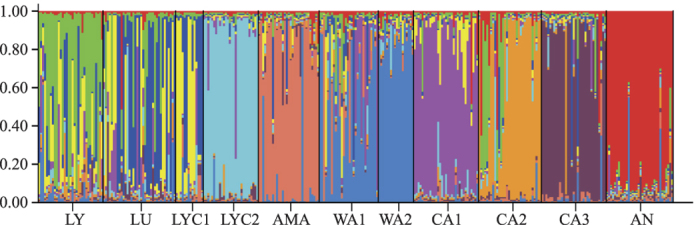
Membership proportion of 323 individuals to ten groups (*K* = 10) according to the Bayesian analysis of population structure carried out with Structure. For abbreviation of populations, see Table 1.

**Figure 3:**
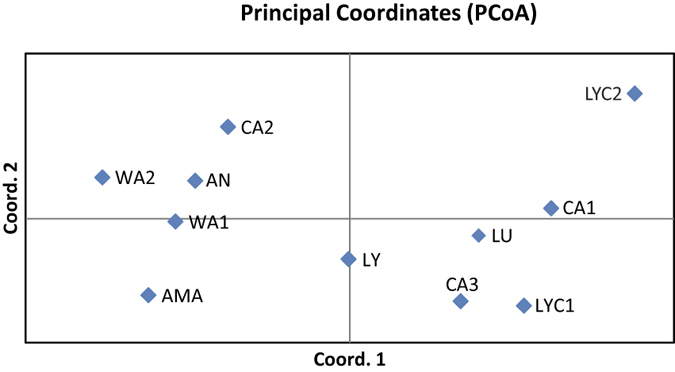
Principal Coordinate Analysis performed from pairwise genetic distances between populations. For abbreviation of populations, see Table 1.

**Figure 4:**
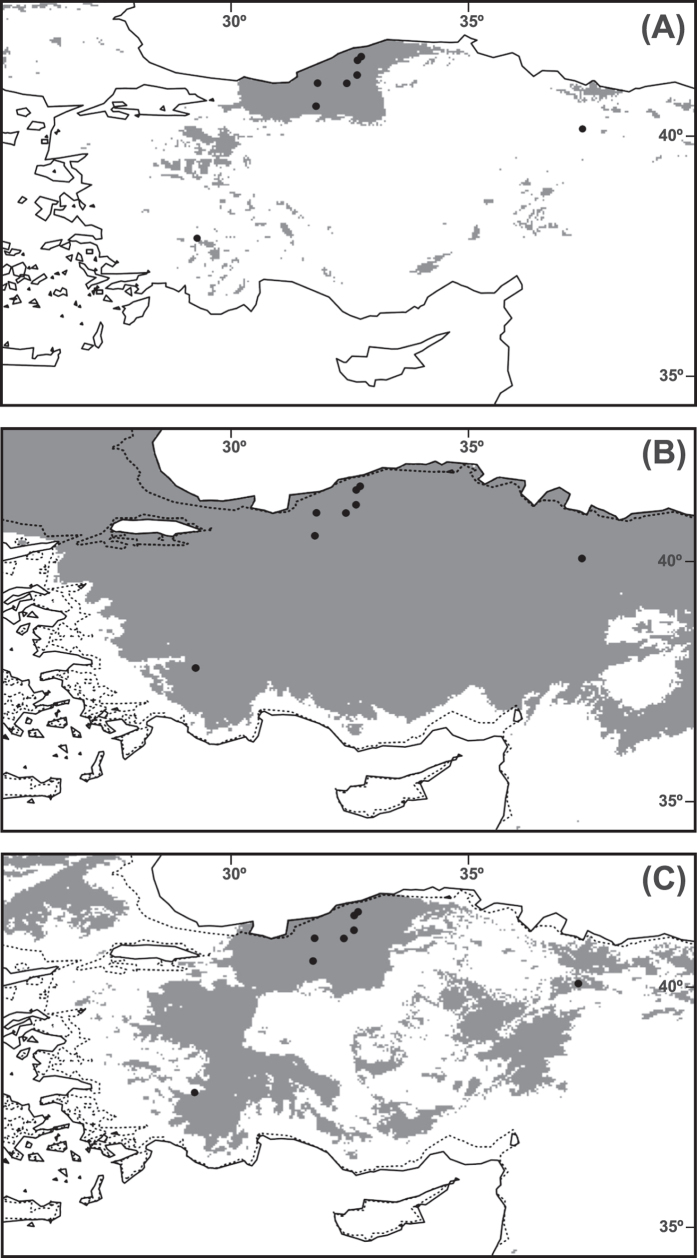
Potential distribution (shaded areas) in Turkey of *Centaurea cadmea* obtained with MaxEnt. (**a**) At the present time; (**b**) at the Last Glacial maximum (LGM, ca. 21,000 years BP) using the Community Climate System Model (CCSM^80^); (**c**) at the LGM using the Model for Interdisciplinary Research on Climate (MIROC^81^). Black dots indicate current populations of the species. The reconstructed LGM coastlines are represented in (**b**) and (**c**), with current coastlines superimposed as dotted lines. This figure has been generated with ArcGIS v. 9.3 (ESRI, Redlands, CA, USA).

Recent gene flow is now almost absent and populations are genetically isolated, as deduced from the results of BayesAss (Table 5). The only case of current flow is the connection between one of the populations of *C. lycia* (LYC1) and *C. luschaniana*. In this case, Barrier results also indicate that there are no barriers between them, even with *B* = 8 (Supplementary Fig. S2). This may be due to lack of topographical barriers that do exist, however, with the other population of *C. lycia* (LYC2; Fig. 1), which grows on the other side of the mountains. The population LYC2 appears to be isolated and free from admixture in Structure (pale blue, Fig. 2) and the Barrier analysis effectively separates it from the rest of populations from *B* = 5 (Supplementary Fig. S2). Other instances of high admixture (*C. lycaonica* and population 1 of *C. wagenitzii*) are evident in Structure (Fig. 2), for which recent gene flow is not a valid explanation as reflected in BayesAss. For these two populations (especially for WA1) we can argue, instead, ancient gene flow as shown by the MIGRATE-N results (Table 6). Although the *Nm* values suggested by this software are not high at all, at least there is some signal of exchange of genes; however, we cannot assign an exact temporal framework for these old contacts, as they may range from the Holocene (~450 ya) to the end of LGM (~18,500 ya) following our previous approximation^15^. According to our reconstruction of the ecological niche at the LGM for one of the species studied here (Fig. 4; see below), gene flow would have been much more extensive during the glaciations than at present.

Lower but still noticeable levels of admixture are evident in almost all species studied (Fig. 2). For these cases, we can also argue ancient gene flow. In none of the cases of admixture, either recent or old, there is morphological evidence of introgression. That is, no intermediate characters have been observed between the species that have been connected. Ancient contacts between non sympatric species in the Mediterranean region have been explained in several cases by altitudinal migrations forced by the glaciations^13^,^47^. In our case, the contacts may have occurred by the pathway of the mountains surrounding the Anatolian plateau (Fig. 1). According to the ENM for *C. cadmea*, its current area is much smaller than the potential distribution of the species in the LGM (Fig. 4C). *Centaurea cadmea* migration would be possible through the zones defined by the ENM, which coincide with the archs of mountains just described, especially via the Anatolian Diagonal in the east of the plateau. At the LGM, these mountainous archs were probably covered by boreal/temperate forests and parkland instead of xeric grasslands or steppe, as revealed by climate simulations^41^,^42^. Fossil pollen records also points towards cold but relatively moist conditions (probably due to the orographic rain) for most of the mountainous southern regions of Anatolia^43^. A pollen record from Lake Iznik, in the mountains of NW Anatolia, is suggestive of steppe but with a still important fraction of arboreal vegetation (ca. 25%)^48^. There are no pollen sites for the Pontic Mountains, but there are currently some relict termophilous species in the region, such as *Pterocarya fraxinifolia*. Along the Black Sea coastline, *Carya* and *Glyptostrobus* persisted until the Holocene, and were probably extinct due to human disturbance^49^. Presence of termophilous species indicates that glacial refugia would have existed in the region. Contrarily, the Anatolian Plateau was dominated by *Artemisia*-steppe; at the only pollen site from the plateau, only 10% of the pollen is arboreal^43^. Presence of steppe suggests much drier conditions to those of the surrounding mountain archs. All the species of the study except *C. wagenitzii* share similar ecological requirements (see Introduction), and it is plausible that the same pathways favorable to *C. cadmea* have been exploited by other species of the study. The lack of barriers between the most isolated species, *C. amaena*, and the core of the SW species in the Taurus (Supplementary Fig. S2) reinforces the hypothesis of the existence of migration routes through the mountains of S Turkey. In addition to the ENM, migration routes though the northern mountain arches are also suggested by the lack of barriers between *C. amaena* and population 3 of *C. cadmea* (Supplementary Fig. S2) as well as by the relatively high *Nm* values between populations 2 and 3 of *C. cadmea* (located in the Pontic Mountains) and some of the studied populations of SW Taurus.

### Greece and Turkey models

A comparison between the diversification patterns in Greece and Anatolia is straightforward. The similarities are relevant, with a main factor having shaped genetic diversity and diversification in *Centaurea* subsect. *Phalolepis*: the rugged and dissected topography. The Anatolian mountains favored the population isolation of a more widespread ancestor of the species of the group, triggering allopatric speciation. At the same time, the mountains would have constituted refugia during the glaciations, protecting the populations from genetic bottlenecks and promoting contacts between them as shown by the high genetic diversity found. Finally, the mountain arch that encircles the Anatolian Plateau, especially the Anatolian Diagonal at the east, offered a pathway during glacials for the migration of species.

The main difference between Greece and Turkey in the diversification of *Centaurea* subsect. *Phalolepis* is the time frame: speciation in Turkey is probably more recent. The main reasons for reaching this conclusion are: (a) Genetic clustering in Greek species using STRUCTURE show only limited level of admixture^15^, while in Turkey the levels of admixture are much higher (Fig. 2). (b) *F*_ST_ values between species are larger in Greece compared to Turkey (0.182 vs. 0.135). These results support that Greek species would have almost achieved complete isolation, while Anatolian ones would be still in progress. In sum, processes involving diversification in both hotspots are basically the same, with a slightly later temporal displacement in Turkey when compared to the Greek case.

### Evolutionary and conservation implications

Mountains elsewhere are often regarded as suitable Quaternary refugia, where both persistence and differentiation of plant lineages would have occurred^6^; thus, it is not surprising that mountains often harbor high taxonomic richness as well significant rates of endemism^50^. For the mountains of the Mediterranean basin, it was suggested a dual model to explain the patterns of endemism^51^: prevalence of paleo-endemics in its western section and dominance of neoendemics in its eastern half; according to these authors, the dominance of differentiation processes over conservative ones in the Eastern Mediterranean basin would have resulted from the relative geologic youth of this sector and, remarkably, the moderate role of Pleistocene glaciations^49^. The present study as well as our previous one^15^ in the subsect. *Phalolepis* of *Centaurea* (a very young group) are paradigmatic of the role of Mediterranean mountains as places where active speciation processes have taken, and are still taking place. The high levels of genetic diversity and the moderate genetic isolation between species support the role of mountains as glacial refugia and “species pumps”.

In conservation biogeography, one of the most debated questions at present is whether priority should be given to areas of active speciation (“species cradles”) or, on the contrary, to areas that are a sink of ancestral taxa (“species museums”)^52^. Although with the conservation of “species museums” we are avoiding the loss of relict taxa (that, in most cases, have unique, irreplaceable evolutionary history), conserving species pumps we are preserving those places that contribute most to the growth of the Tree of Life (i.e., we are guaranteeing the maximum levels of both present and future biodiversity). Conserving the eastern Mediterranean mountains in a more or less pristine state is, however, a challenging issue; overgrazing and erosion are rampant, and tourism impacts are quickly increasing^53^. Regrettably only 1.2% of Turkey’s terrestrial area is strictly protected (much below the international standards), and high mountains, shrublands, and steppe ecosystems are underrepresented^53^. Therefore, although conserving large landscapes is perhaps the best tool in plant by preserving processes that create and maintain biodiversity^54^, it seems more realistic to ensure the conservation at population level of those species of conservation interest, such as narrow endemics. Unfortunately, most of the studied species do not enjoy at present of suitable conservation measures (even despite that some of them are classified as CR); for instance, less than 20% of all the populations of the studied species are located within a nature reserve and, to our knowledge, no specific conservation measures, either *in-situ* or *ex-situ*, exist.

## Methods

### Plant material

Most species of subsect. *Phalolepis* clasically recognized^11^ are included in the study, with the addition of a couple of taxa described afterwards. We excluded *C. hieropolitana* and *C. tossiensis* (see Introduction). Tetraploid *C. aphrodisea* and *C. dursunbeyensis* were also excluded due to the difficulties in interpreting microsatellite results in polyploids^55^. Thereafter, our survey includes seven species: *C. amaena* (one population), *C. antalyensis* (one population), *C. cadmea* (three populations), *C. luschaniana* (one population), *C. lycaonica* (one population), *C. lycia* (two populations) and *C. wagenitzii* (two populations).

*Centaurea amaena* grows in rocky places in the Kayseri province and it is restricted to a small area between Yılanlı and Erciyes Mountain. The only two localities together cover an area of around 0.55 km^2^, and the number of individuals was calculated to be 5672. Its threat category was suggested to be EN (Endangered)^56^. However, the species displays fairly local distribution in an isolated area open to urbanisation as well as limited number of mature individuals. The species should be categorised as CR (Critically Endangered) according to 2001 IUCN criteria^57^ [CR B1ab(i,iii,iv) + 2ab(i,iii,iv)].

*Centaurea antalyensis* is known only from the type locality and the number of mature individuals is below 250. The species was listed as CR (Critically Endangered)^58^. According to our observations, the species is very local and its distribution area is less than 10 km^2^, which agrees with the assigned category.

*Centaurea cadmea* is represented by two subspecies in Turkey. Both of them have fairly local distribution limited to one or two small populations. *Centaurea cadmea* subsp. *cadmea* is known from a few localities in Denizli, growing on calcareous rocky slopes. The number of individuals is less than 250 and this subspecies is under high risk due to overgrazing. It was evaluated as LR (Lower Risk)^56^. However, it should be categorized as Critically Endangered (CR) because of its local distribution [B1ab(i,iv,v)]. *Centaurea cadmea* subsp. *pontica* is present in the Zonguldak and Bartın provinces, where it is common. It grows on rocky slopes overlooking deep valleys, at 275–700 m. Its threat category should be VU following 2001 IUCN criteria^57^ [B2ab(i,iii,iv)].

*Centaurea luschaniana* is a local endemic species that is restricted to a limited area between Elmalı and Korkuteli. This species is known from 4 or 5 populations on the line Korkuteli-Elmalı. Its threat category was evaluated before as LR (Lower Risk)^56^. According to our observations, the populations of this species are in good health. However, some of them may become under intense pressure due to farming activities of nearby villages. Therefore, we suggest that the species should be categorised as VU (Vulnerable) following 2001 IUCN criteria^57^ [VU B1ab(ii,iv)].

*Centaurea lycaonica* is a local endemic species in Konya province of Central Anatolia. It was classified as Critically Endangered (CR)^56^. There are four–five very close populations that are threatened due to extreme livestock grazing and gold mining^59^. Further alteration of the habitat of *C. lycaonica* would result in the extinction of the species. Thereafter, the species is correctly categorized as CR (Critically Endangered) based on 2001 IUCN criteria^57^ [B1ab(i,ii,iii,v) + 2ab(ii,iv,v)].

*Centaurea lycia* is a regional endemic species growing on rocky cliffs in Antalya province. There are six populations, which appear in good condition in terms of mature individuals (K. Ertuğrul, pers. obs.). Its threat category was evaluated as LR (Lower Risk)^56^. However, the species is exposed to heavy tourism activities as well as suffering the negative impacts of urbanisation (K. Ertuğrul pers. obs.). Therefore, we suggest that the species should be categorised as Vulnerable (VU) following 2001 IUCN criteria^57^ [VU B1ab(ii,iii,iv)].

*Centaurea wagenitzii* is known only from the type locality, with an estimated area of occupancy of less than 5 km^2^. Its threat category was evaluated before as EN (Endangered)^56^ under the criterion B2a. According to our observations, the population comprises less than 250 individuals, and it should be classified as Critically Endangered (CR) based on the 2001 IUCN criteria^57^ [B2ab(i,ii,iv,v)].

Voucher information is provided in Supplementary Table S4, and a map with the location of the collected populations is shown in Fig. 1.

### DNA isolation and microsatellite loci

We extracted genomic DNA from dried leaves using the CTAB method^60^. For verifying cross-amplification of the seven Turkey-endemic species, we carried out a preliminary test using 16 SSR markers developed for other species of *Centaurea* (*C. corymbosa*^61^, *C. diffusa* and *C. stoebe*^62^). We amplified polymorphic bands from seven microsatellites (*CD37, 42CM27, 12B1, 13D10, 17E3, 21D9, 28A7*) in all the studied species, as explained in López-Vinyallonga *et al*.^15^. We used at least 30 individuals per population when possible, accounting for a total of 323 individuals from 11 populations. Genotyping was performed as described in López-Vinyallonga *et al*.^15^.

### Genetic analysis

Software GenAlEx v. 6.1 63 and Genetix v. 4.05 64 were used for estimating genetic diversity parameters at both species and population levels: (a) mean number of alleles per locus (*A*); (b) number of private alleles (*PA*); (c) percentage of polymorphic loci (*P*_95_); (d) observed heterozygosity (*H*_o_); (e) unbiased expected heterozygosity (*H*_e_); and (f) inbreeding coefficient (*F*_IS_) by the method of Weir & Cockerham^65^. GenePop v. 4.0.10^66^ was used for calculating possible deviations from Hardy-Weinberg (H–W) equilibrium and for checking genotypic linkage disequilibrium between pairs of loci at population level and across all populations; for both calculations the software uses a Fisher’s exact test following the Markov chain (MC) algorithm^67^. Frequency of null alleles was calculated using FreeNA^68^, which was also used to estimate *F*_ST_ values between pairs of populations and species (with and without the ENA correction for null alleles^68^).

The spatial genetic structure was assessed through four different methods. First, Structure v. 2.3.4^69^, a widely-employed clustering software that is a based on a Bayesian algorithm, was used. On the basis of preliminary runs, *K* was run from 1 to 12 (20 iterations per *K*) assuming an admixture model with correlated allele frequencies, and with a priori grouping of individuals into populations (but not into species). The length of burn-in period and the MCMC replications were set to 10^5^ and 10^6^, respectively. The most likely value of *K* was determined both by choosing the smallest *K* after the log probability of data [ln Pr(*X*|*K*)] values reached a plateau^69^ and by the Δ*K* statistic of Evanno *et al*.^70^ with the aid of Structure Harvester^71^. Second, a molecular variance analysis (AMOVA) was run with the aid of GenAlEx v. 6.1, establishing two hierarchical levels: (i) among taxonomic groups (species), (ii) among populations, and within populations. Third, a Principal Coordinates Analysis (PCoA) at population level was carried out with the same software (GenAlEx). Fourth, putative genetic barriers between populations were detected with the software Barrier v. 2.2^72^; significance of barriers was tested by bootstraping 1000 *D*_A_^73^ matrices that were previosuly obtained with Microsatellite Analyzer (MSA) v. 4.05 software^74^.

Gene flow was estimated with two time-frameworks. First, we estimated recent (i.e. within the recent 2–3 generations) migration rates between individual populations with the sofware BayesAss v. 1.3^75^. As program settings, the default values were used (MCMC iterations, 3 × 10^6^; length of the burn-in, 999,999; sampling frequency, 2000; delta value, 0.15). Second, historical mutation-scaled migration rates (*M* = *m/μ*, where *m* is migration rate and *μ* is mutation rate per generation) were estimated using MIGRATE-N v. 3.6.4^76^. Ten replicates were run under a Brownian motion model, assuming constant mutation rate for all loci. With a Bayesian approach, a long chain with 20,000 genealogies to sample was run, with a sampling increment of 100 (thus, totalling 2,000,000 genealogies for each replicate); the burn-in was set to 20,000. A static heating scheme was chosen (temperatures were specified to 1.00, 1.50, 3 and 1 × 10^6^), with uniform prior distribution both for *Θ* and *M* (min: 0; max: 100; delta: 10). The effective number of migrants per generation (*Nm*) among populations was estimated using the formula 4*Nm* = *ΘM* 76. We could not estimate migration rate (*m*) values, provided that those obtained from MIGRATE-N are mutation-scaled (*M*), and mutation rates (*μ*) for microsatellites for the genus *Centaurea* are not available. Total immigration and emigration rates for each population were obtained by summing values of *Nm*. Analyses were carried out at the CIPRES bioinformatic facility^77^.

### Ecological niche modelling

Ecological niche modelling (ENM) was performed to evaluate the potential distribution of the Turkey species of *Centaurea* under present climatic conditions and to project it to the Last Glacial Maximum (LGM, ca. 21,000 yr BP). The ENM was only performed with two of the study species (*C. cadmea* and *C. luschaniana*) because for the other taxa the number of wild occurrences was not enough to get reliable models (<5)^78^. We employed the maximum entropy algorithm, as implemented in MaxEnt v. 3.3 79. The current distribution information for both *Centaurea* species was obtained from presence records included in the Global Biodiversity Information Facility (www.gbif.org), from literature^12^,^14^, and from the sampling sites of this study. In total, after removing duplicate records within each pixel (2.5 arc-min, *ca*. 5 km), we obtained 8 and 5 presence records for *C. cadmea* and *C. luschaniana*, respectively. A set of 19 bioclimatic variables at 2.5 arc-min resolution covering the distribution range (and neighboring areas) for both species under current conditions (1950–2000) were downloaded from the WorldClim website (www.worldclim.org). After a correlation analysis in a random sample of 10,000 points within the study area, we selected a smaller set of nine (relatively) uncorrelated (*r* ≥ |0.9|) variables: isothermality (bio3), temperature seasonality (bio4), mean temperature of the wettest quarter (bio8), mean temperature of the warmest quarter (bio10), mean temperature of the coldest quarter (bio11), annual precipitation (bio12), precipitation of the driest month (bio14), precipitation seasonality (bio15), and precipitation of the coldest quarter (bio19). The selection of variables from pairs or groups of highly correlated ones was done on the basis of their relative contribution to the model (percent contribution, jackknife tests of variable importance). The distribution model under current conditions was projected to the LGM using palaeoclimatic layers simulated by both the Community Climate System Model (CCSM)^80^ and the Model for Interdisciplinary Research on Climate (MIROC)^81^. Replicate runs (10) of MaxEnt (using the “bootstrap” method) were performed to ensure reliable results.

Model testing when the number of occurrences is small (<25) can be problematic, because the “training” and “test” datasets would be too low^78^. To overcome this, we used a methodology based on a jackknife (or “leave-one-out”) procedure^78^ to test the model. With this procedure the model is built (or “trained”) using *n* − 1 occurrences, and tested using the discarded locality (that is, by evaluating the ability to predict the single locality excluded from the training dataset). Thus, eight and five predictions were obtained for *C. cadmea* and *C. luschaniana*, respectively. We used the “lowest presence threshold” (LPT, also commonly referred as “minimum training presence” in the MaxEnt terminology) as the cut-off value to decide whether the discarded locality is “suitable” or “unsuitable”. The LPT is considered as more conservative than other approaches (such as the 10% fixed threshold) since identifies the minimum predicted area possible whilst maintaining zero omission error in the training data set^78^. Performance of models for both *C. cadmea* and *C. luschaniana* was evaluated through success rate (*q*, which is the proportion of right predictions) and statistical significance (a *P*-value computed across the set of jackknife predictions), which was done using the software provided by Pearson *et al*.^78^. The jackknife analysis indicated that the model was successful at predicting known occurrences as suitable areas for *C. cadmea* (success rate = 75%, *P* < 0.001) but not for *C. luschaniana* (success rate = 40%, *P* = 0.041). Thus, definitive ENM models (that is, using all occurrence points) for the present and LGM were only built for *C. cadmea*. Before it, however, we carried out tuning experiments varying the level of regularization, in order to improve the performance of the model^82^. After discarding regularization multipliers >2 (as they diminished AUC values considerably according to exploratory runs), leave-one-out models were built with up to eight regularization multipliers (0.25, 0.50, 0.75, 1.00, 1.25, 1.50, 1.75, and 2.00). Based on the optimal combination of AUC values and success rates (*q*)^83^, the best model was that using a regularization multiplier of 0.5 (Supplementary Fig. S4).

To convert continuous suitability values obtained for each pixel to presence/absence (that is, a binary map), we chose the minimum training presence value as the threshold. Finally, a jackknife analysis was used to evaluate the relative importance of the nine bioclimatic variables employed. All ENM predictions were visualized in ArcGIS v. 9.3 (ESRI, Redlands, CA, USA), with the aid of Hawth’s Analysis Tools^84^.

## Additional Information

**How to cite this article**: López-Pujol, J. *et al*. Speciation and genetic diversity in *Centaurea* subsect. *Phalolepis* in Anatolia. *Sci. Rep.* **6**, 37818; doi: 10.1038/srep37818 (2016).

**Publisher's note:** Springer Nature remains neutral with regard to jurisdictional claims in published maps and institutional affiliations.

## Supplementary Material

Supplementary Information

## Figures and Tables

**Table 1:** Main parameters of genetic diversity for each population computed after the seven polymorphic loci.

Species/populations^1^	Coordinates	*N*	*A*	*PA*	*P*_95_	*H*_o_	*H*_e_	*F*_IS_
***C. lycaonica***	N37° 45.054′ E32° 04.529′							
**LY**		**33**	**5.571**	**3**	**0.857**	**0.269**	**0.498**	0.466*
***C. luschaniana***	N36° 56.681′ E30° 09.617′							
**LU**		**37**	**7.286**	**5**	**1.000**	**0.346**	**0.582**	0.409*
***C. lycia***								
LYC1	N37° 00.045′ E30° 29.409′	14	4.286	1	**0.857**	0.350	0.547	0.373*
LYC2	N36° 53.511′ E30° 22.153′	28	4.286	0	1.000	0.468	0.491	0.048
**Mean**		**21**	**4.286**	**0.5**	**0.929**	**0.409**	**0.519**	**0.211**
Species level		42	6.429	1	1.000	0.428	0.580	0.266*
***C. amaena***	N38° 42.874′ E35° 25.064′							
**AMA**		**31**	**5.857**	**1**	**1,000**	**0.317**	**0.615**	0.491*
***C. wagenitzii***								
WA1	N36° 17.817′ E30° 28.455′	30	6.429	2	1.000	0.485	0.669	0.280*
WA2	N36° 18.770′ E30° 27.812′	18	4.286	1	1.000	0.671	0.618	-0.088
**Mean**		**24**	**5.358**	**1.5**	**1.000**	**0.578**	**0.644**	**0.096**
Species level		48	7.571	4	1.000	0.559	0.663	0.158*
***C. cadmea***								
CA1	N37° 45.052′ E29° 15.995′	33	5.429	2	1.000	0.517	0.575	0.103*
CA2	N41° 5.752′ E31° 48.982′	32	4.286	1	1.000	0.439	0.522	0.160*
CA3	N41° 35.356′ E32° 41.110′	33	5.714	3	**0.857**	0.355	0.546	0.355*
**Mean**		**32.7**	**5.143**	**2**	**0.952**	**0.437**	**0.548**	**0.206**
Species level		98	9.143	7	1.000	0.436	0.660	0.340*
***C. antalyensis***	N36° 54.493′ E31° 48.939′							
**AN**		**34**	**6.000**	**4**	**1.000**	**0.648**	**0.655**	**0.011**
**Mean (11 populations)**		**29.4**	**5.643**	**2.1**	**0.961**	**0.430**	**0.580**	**0.237**

*N*, sample size; *A*, mean number of alleles per locus; *PA*, number of private alleles; *P*_95_, percentage of polymorphic loci (95% criterion); *H*_o_, observed heterozygosity; *H*_e_, unbiased expected heterozygosity; *F*_IS_, inbreeding coefficient; ^*^P < 0.05.

^1^Populations are identified by an alphanumeric code. See Supplementary Table S4 for more details on the sampled localities.

**Table 2:** Pairwise comparisons showing differentiation between populations based on *F*_ST_.

	LY	LU	LYC1	LYC2	AMA	WA1	WA2	CA1	CA2	CA3	AN
LY	0.000										
LU	0.057*	0.000									
LYC1	0.122*	0.027	0.000								
LYC2	0.287*	0.201*	0.266*	0.000							
AMA	0.212*	0.190*	0.183*	0.297*	0.000						
WA1	0.157*	0.121*	0.144*	0.257*	0.118*	0.000					
WA2	0.217*	0.187*	0.196*	0.285*	0.176*	0.046*	0.000				
CA1	0.163*	0.133*	0.179*	0.197*	0.243*	0.170*	0.214*	0.000			
CA2	0.225*	0.230*	0.276*	0.254*	0.229*	0.184*	0.206*	0.232*	0.000		
CA3	0.203*	0.152*	0.167*	0.281*	0.144*	0.196*	0.248*	0.219*	0.274*	0.000	
AN	0.206*	0.171*	0.202*	0.260*	0.186*	0.156*	0.143*	0.206*	0.214*	0.198*	0.000

^*^P < 0.05.

**Table 3:** Pairwise comparisons showing differentiation between species based on *F*_ST_.

	LY	LU	LYC	AMA	WA	CA	AN
LY	0.000						
LU	0.057*	0.000					
LYC	0.178*	0.095*	0.000				
AMA	0.212*	0.190*	0.217*	0.000			
WA	0.163*	0.135*	0.176*	0.133*	0.000		
CA	0.095*	0.086*	0.086*	0.123*	0.108*	0.000	
AN	0.206*	0.171*	0.196*	0.186*	0.141*	0.130*	0.000

^*^P < 0.05.

**Table 4:** Analysis of the molecular variance (AMOVA).

Source	*df*	SS	Variance component	% variation
Among taxa	6	169.483	0.001	0.05
Among Populations	4	98.132	0.418	15.53*
Within Populations	635	1442.877	2.272	84.42*

^*^P < 0.05.

**Table 5:** Mean recent migration rates (*m*) among the studied populations, estimated from seven microsatellite loci using the BayesAss program.

To	From										
	LY	LU	LYC1	LYC2	AMA	WA1	WA2	CA1	CA2	CA3	AN
LY	0.985 (0.949–1.000)	0.004 (0.000–0.028)	0.008 (0.000–0.051)	0.001 (0.000–0.010)	0.002 (0.000–0.016)	0.003 (0.000–0.025)	0.002 (0.000–0.018)	0.001 (0.000–0.009)	0.001 (0.000–0.013)	0.002 (0.000–0.016)	0.001 (0.000–0.011)
LU	0.002 (0.000–0.018)	0.980 (0.922–1.000)	**0.241 (0.133–0.317)**	0.001 (0.000–0.011)	0.002 (0.000–0.016)	0.002 (0.000–0.013)	0.002 (0.000–0.019)	0.001 (0.000–0.011)	0.001 (0.000–0.010)	0.002 (0.000–0.017)	0.001 (0.000–0.009)
LYC1	0.001 (0.000–0.010)	0.001 (0.000–0.011)	0.687 (0.667–0.733)	0.001 (0.000–0.012)	0.002 (0.000–0.014)	0.001 (0.000–0.014)	0.002 (0.000–0.019)	0.001 (0.000–0.012)	0.001 (0.000–0.010)	0.001 (0.000–0.012)	0.001 (0.000–0.011)
LYC2	0.001 (0.000–0.008)	0.002 (0.000–0.014)	0.008 (0.000–0.050)	0.989 (0.961–1.000)	0.002 (0.000–0.014)	0.002 (0.000–0.016)	0.003 (0.000–0.023)	0.001 (0.000–0.010)	0.001 (0.000–0.011)	0.001 (0.000–0.011)	0.001 (0.000–0.010)
AMA	0.001 (0.000–0.009)	0.001 (0.000–0.011)	0.008 (0.000–0.051)	0.001 (0.000–0.012)	0.968 (0.923–0.998)	0.003 (0.000–0.023)	0.003 (0.000–0.026)	0.001 (0.000–0.011)	0.001 (0.000–0.008)	0.001 (0.000–0.012)	0.001 (0.000–0.011)
WA1	0.001 (0.000–0.011)	0.002 (0.000–0.014)	0.009 (0.000–0.057)	0.001 (0.000–0.012)	0.009 (0.000–0.040)	0.980 (0.928–1.000)	0.003 (0.000–0.023)	0.001 (0.000–0.010)	0.001 (0.000–0.012)	0.003 (0.000–0.020)	0.001 (0.000–0.011)
WA2	0.001 (0.000–0.010)	0.002 (0.000–0.015)	0.007 (0.000–0.048)	0.001 (0.000–0.008)	0.004 (0.000–0.026)	0.002 (0.000–0.020)	0.976 (0.910–0.999)	0.001 (0.000–0.011)	0.001 (0.000–0.012)	0.002 (0.000–0.016)	0.001 (0.000–0.011)
CA1	0.002 (0.000–0.015)	0.004 (0.000–0.030)	0.008 (0.000–0.047)	0.001 (0.000–0.010)	0.002 (0.000–0.015)	0.002 (0.000–0.020)	0.002 (0.000–0.021)	0.989 (0.961–1.000)	0.001 (0.000–0.011)	0.002 (0.000–0.013)	0.001 (0.000–0.011)
CA2	0.002 (0.000–0.019)	0.002 (0.000–0.012)	0.008 (0.000–0.050)	0.001 (0.000–0.012)	0.004 (0.000–0.021)	0.002 (0.000–0.014)	0.002 (0.000–0.021)	0.001 (0.000–0.010)	0.988 (0.956–1.000)	0.002 (0.000–0.016)	0.001 (0.000–0.010)
CA3	0.001 (0.000–0.012)	0.002 (0.000–0.013)	0.007 (0.000–0.045)	0.001 (0.000–0.011)	0.003 (0.000–0.020)	0.002 (0.000–0.015)	0.002 (0.000–0.016)	0.001 (0.000–0.012)	0.001 (0.000–0.011)	0.981 (0.941–0.999)	0.002 (0.000–0.012)
AN	0.001 (0.000–0.012)	0.002 (0.000–0.013)	0.007 (0.000–0.046)	0.001 (0.000–0.011)	0.002 (0.000–0.016)	0.001 (0.000–0.014)	0.002 (0.000–0.023)	0.001 (0.000–0.009)	0.001 (0.000–0.011)	0.002 (0.000–0.015)	0.988 (0.957–1.000)

Values on the diagonal (underlined) indicate the proportion of individuals in each generation that are not migrants. Simulations in BayesAss show that in instances where there is no information in the data, the mean *m* and 95% confidence interval for data sets of 11 populations are 0.017 and 0.000–0.114, respectively; values in bold are the *m* rates that are informative.

**Table 6:** Median historical gene flow (*Nm*) among the studied populations, estimated from seven microsatellite loci using the formula 4*Nm* = *ΘM* (with *M* values obtained with MIGRATE-N).

To	*Θ*	From											
		LY	LU	1LYC	2LYC	AMA	1WA	2WA	1CA	2CA	3CA	AN	Total *Nm* (as immigration rates)
LY	0.900 (0–2.000)		0.428 (0–0.855)	0.473 (0–0.900)	0.293 (0–0.630)	0.518 (0–1.260)	0.383 (0–0.810)	0.473 (0–0.945)	0.383 (0–0.765)	0.608 (0–1.215)	0.428 (0–0.810)	0.383 (0–0.855)	4.365
LU	0.900 (0–1.800)	0.383 (0–0.810)		0.473 (0–0.990)	0.293 (0–0.585)	0.293 (0–0.585)	0.428 (0–0.945)	0.428 (0–0.855)	0.338 (0–0.630)	0.338 (0–0.675)	0.338 (0–0.810)	0.293 (0–0.585)	3.600
1LYC	0.700 (0–1.800)	0.578 (0–1.785)	0.648 (0–2.415)		0.683 (0–1.610)	0.368 (0–1.155)	0.753 (0.070–1.470)	0.298 (0–0.735)	0.508 (0–1.155)	0.368 (0–1.120)	0.928 (0–2.835)	0.368 (0–0.805)	5.495
2LYC	0.700 (0–1.800)	0.368 (0–0.735)	0.473 (0–1.225)	0.368 (0–0.700)		0.368 (0–0.805)	0.438 (0–0.875)	0.403 (0–0.980)	0.543 (0–1.050)	0.718 (0.070–1.260)	0.403 (0–0.735)	0.298 (0–0.665)	4.375
AMA	0.700 (0–1.800)	0.263 (0–0.595)	0.368 (0–0.770)	0.508 (0–0.945)	0.368 (0–0.770)		0.298 (0–0.630)	0.368 (0–0.735)	0.368 (0–0.875)	0.333 (0–0.700)	0.263 (0–0.595)	0.333 (0–0.805)	3.465
1WA	0.900 (0–2.000)	0.608 (0–1.575)	0.923 (0–1.665)	0.653 (0–1.305)	0.518 (0–1.395)	0.608 (0–1.440)		1.193 (0.225–2.385)	0.518 (0–1.125)	0.518 (0–1.080)	0.383 (0–0.855)	0.383 (0–0.855)	6.300
2WA	0.700 (0–1.800)	0.333 (0–1.015)	0.963 (0.070–1.890)	0.823 (0.035–1.540)	0.858 (0.070–2.170)	0.543 (0–1.505)	1.068 (0–3.010)		0.928 (0.105–1.890)	1.068 (0–1.820)	0.578 (0–1.155)	0.648 (0–1.820)	7.805
1CA	0.900 (0–1.800)	0.473 (0–0.900)	0.473 (0–1.080)	0.518 (0–1.125)	0.383 (0–0.720)	0.383 (0–0.765)	0.428 (0–0.945)	0.608 (0–1.215)		0.383 (0–0.810)	0.518 (0–1.035)	0.473 (0–1.080)	4.635
2CA	0.700 (0–1.800)	0.333 (0–0.700)	0.473 (0–0.875)	0.403 (0–0.805)	0.508 (0–1.015)	0.333 (0–0.665)	0.333 (0–0.665)	0.508 (0–0.980)	0.368 (0–0.700)		0.368 (0–0.700)	0.298 (0–0.700)	3.920
3CA	0.700 (0–1.800)	0.368 (0–0.875)	0.508 (0–0.945)	0.333 (0–0.840)	0.333 (0–0.700)	0.333 (0–0.630)	0.368 (0–0.700)	0.473 (0–0.910)	0.473 (0–0.945)	0.298 (0–0.665)		0.403 (0–0.875)	3.885
AN	0.900 (0–2.000)	0.338 (0–0.720)	0.338 (0–0.675)	0.293 (0–0.585)	0.338 (0–0.720)	0.428 (0–0.765)	0.338 (0–0.720)	0.338 (0–0.675)	0.338 (0–0.675)	0.383 (0–0.540)	0.338 (0–0.720)		3.465
Total *Nm* (as emigration rates)		4.040	5.590	4.840	4.570	4.170	4.830	5.085	4.760	5.010	4.540	3.875	

As suggested by Beerli (2006), the median is used instead of the mean since the latter is heavily influenced by outliers. In parentheses, 95% confidence interval. *Θ* is the mutation-scaled effective population size, as obtained from MIGRATE-N.
