# Supplementary material for: Speciation and genetic diversity in Centaurea subsect. Phalolepis in Anatolia
Source: Sci Rep. 2016 Nov 25;6:37818. doi: 10.1038/srep37818 (PMC5122891; doi:10.1038/srep37818)
Supplement: Supplementary Information [file srep37818-s1.pdf]

Supplementary information

Speciation and genetic diversity in *Centaurea* subsect. *Phalolepis* in Anatolia

Jordi López-Pujol[1], Sara López-Vinyallonga[1], Alfonso Susanna[1], Kuddisi Ertuğrul[2], Tuna Uysal[2], Osman Tugay[2], Arbi Guetat[3] & Núria Garcia-Jacas[1*]

[1]Botanic Institute of Barcelona (IBB-CSIC-ICUB), Pg. del Migdia, s/n, ES-08038 Barcelona, Spain
[2] Faculty of Science and Art, Selcuk University, TR-42031 Konya, Turkey
[3] Department of Biology, College of Sciences, Northern Border University, Arar, Saudi Arabia

Correspondence and requests for materials should be addressed to N.G.-J. (email: ngarciajacas@ibb.csic.es).

Table S1. Pairwise comparisons showing differentiation between populations based on F_{ST}, using the ENA correction with the software FreeNA. *P< 0.05.

	LY	LU	LYC1	LYC2	AMA	WA1	WA2	CA1	CA2	CA3	AN
LY	0.000										
LU	0.046*	0.000									
LYC1	0.113*	0.035*	0.000								
LYC2	0.270*	0.193*	0.251*	0.000							
AMA	0.187*	0.161*	0.171*	0.281*	0.000						
WA1	0.133*	0.105*	0.141*	0.250 *	0.105*	0.000					
WA2	0.202*	0.180*	0.197*	0.282 *	0.177*	0.054*	0.000				
CA1	0.151*	0.129*	0.178*	0.203 *	0.233*	0.162*	0.212*	0.000			
CA2	0.206*	0.213*	0.253*	0.239*	0.222*	0.178*	0.206*	0.223*	0.000		
CA3	0.176*	0.134*	0.150*	0.262 *	0.119*	0.171*	0.235*	0.207*	0.249*	0.000	
AN	0.196*	0.162*	0.197*	0.250*	0.173*	0.151*	0.138*	0.201*	0.207*	0.182*	0.000

Table S2. Pairwise comparisons showing differentiation between species based on F_{ST}, using the ENA correction with the software FreeNA. *P< 0.05.

	LY	LU	LYC	AMA	WA	CA	AN
LY	0.000						
LU	0.050*	0.000					
LYC	0.160*	0.083*	0.000				
AMA	0.187*	0.161*	0.192*	0.000			
WA	0.139*	0.116*	0.158*	0.121*	0.000		
CA	0.083*	0.072*	0.074*	0.109*	0.098*	0.000	
AN	0.196*	0.162*	0.184*	0.173*	0.135*	0.125*	0.000

Table S3. Levels of genetic diversity and genetic differentiation in the investigated diploid *Centaurea* taxa, as detected with microsatellites. *N*, Number of studied populations; *A*, mean number of alleles per locus; H_e, unbiased expected heterozygosity.

Taxon	*N*	Geographical range*	IUCN threat category**	*A*	H_e	Reference
C. aeolica Lojac. subsp. *aeolica*	1	Narrow endemic	Unknown/Not listed	—	0.073	Guarino *et al.*[1]
C. amaena Boiss. & Balansa	1	Narrow endemic	CR	5.857	0.615	This study
C. antalyensis H. Duman & A. Duran	1	Narrow endemic	CR	6.000	0.655	This study
C. aspera L.	9	Widespread	Not listed	5.330	0.550	Ferriol *et al.*[2]
C. brunnea (Halácsy) Halácsy	2	Narrow endemic	Not listed	4.063	0.601	López-Vinyallonga *et al.*[3]
C. busambarensis Guss.	1	Narrow endemic	Unknown/Not listed	—	0.383	Guarino *et al.*[1]
C. cadmea subsp. *cadmea* Boiss.	1	Endemic	CR	5.429	0.575	This study
C. cadmea subsp. *pontica* Wagenitz	2	Endemic	VU	5.000	0.534	This study
C. cineraria L. subsp. *cineraria*	9	Endemic	Unknown/Not listed	—	0.269	Guarino *et al.*[1]
C. corymbosa Pourr.	6	Narrow endemic	VU	—	0.502	Fréville *et al.*[4]
C. chrysocephala Phitos & T. Georgiadis	5	Endemic	Not listed	5.325	0.609	López-Vinyallonga *et al.* [3]
C. deusta Ten.	2	Widespread	Unknown/Not listed	5.313	0.605	López-Vinyallonga *et al.* [3]
C. diffusa Lam.	13	Widespread	Unknown/Not listed	5.831	0.514	Marrs *et al.*[5]
C. emigrantis Bubani	4	Endemic	LC	5.042	0.560	López-Alvarado[6]
C. gymnocarpa Moris & De Not.	1	Narrow endemic	VU	—	0.365	Guarino *et al.*[1]
C. heldreichii Halácsy	1	Narrow endemic	CR	3.875	0.412	López-Vinyallonga *et al.*[3]
C. horrida Badarò	7	Narrow endemic	EN	—	0.779	Mameli *et al.*[7]
C. leucadea Lacaita	1	Narrow endemic	CR	—	0.349	Guarino *et al.*[1]
C. litochorea T. Georgiadis & Phitos	2	Narrow endemic	VU	6.938	0.655	López-Vinyallonga *et al.*[3]
C. luschaniana Heimerl ex Stapf	1	Endemic	VU	7.286	0.582	This study
C. lycaonica Boiss. & Heldr.	1	Narrow endemic	CR	5.571	0.498	This study
C. lycia Boiss.	2	Endemic	VU	4.286	0.519	This study
C. messenicolasiana T. Georgiadis, Dimitrellos & Routsi	1	Narrow endemic	VU	5.375	0.587	López-Vinyallonga *et al.*[3]
C. princeps Boiss. & Heldr.	2	Narrow endemic	EN	4.563	0.569	López-Vinyallonga *et al.*[3]
C. stoebe L. subsp. *stoebe*	2	Widespread	Unknown/Not listed	3.100	0.466	Marrs *et al.*[8]
C. tripontina López-Alvarado, L. Sáez, Filigh., Guardiola & Susanna	2	Narrow endemic	EN	3.667	0.440	López-Alvarado[6]
C. panormitana Lojac. subsp. *ucriae* (Lacaita) Greuter	1	Narrow endemic	Unknown/Not listed	—	0.143	Guarino *et al.*[1]

C. veneris (Sommier) Bég.	1	Narrow endemic	Unknown/Not listed	—	0.567	Guarino *et al.*[1]
C. wagenitzii Hub.-Mor.	2	Narrow endemic	CR	5.358	0.644	This study
**Mean**	**3.0**	**—**	**—**	**5.160**	**0.504**	**—**

*Narrow endemic, taxa that are restricted to one or very few localities, occupying a very small area (few km^2), or with low total population sizes (<10,000); endemic, taxa with a limited number of localities (<20) and with limited distribution areas (<10,000 km^2), widespread, with a greater number of localities or wider distribution areas.

**According to the most recent categories and criteria published by the IUCN[9]. Categories were obtained from the references listed in the rightmost column, or from regional or local red lists and red books.

Table S4. Voucher information for the seven species investigated. Taxon name, origin of the material with herbaria, acronym of population and coordinates

Species	Plant Locality	Coordinates
Centaurea amaena Boiss. & Balansa	B5 Kayseri: Yılan Dağı, Kayalık slopes, 1141 m, *Ertuğrul, Tugay*, *Uysal* & *Susanna 2721*, 23.vi.2009 (BC) **[AMA]**	N38° 42.874' E35° 25.064'
Centaurea antalyensis H. Duman & A. Duran	C3 Antalya: Akseki, between Murtiçi and Güzelsu, 1036 m, *Ertuğrul*, *Tugay*, *Uysal* & *Susanna 2729*, 25.vi.2009 (BC) **[AN]**	N36° 54.493' E31° 48.939'
Centaurea cadmea Boiss.	C2 Denizli: Honaz, on the castle, 689 m, *Ertuğrul, Tugay*, *Uysal* & *Susanna 2708*, 20.vi.2009 (BC) **[CA1]**<br>A4 Zonguldak: Devrek, Eğerci, Taşlık village, 200 m, 03.vi.2009, *Tugay 5761* (KNYA) **[CA2]**<br>A4 Bartın: Ulus-Ulukaya Şelaesi, Kaya road, 241 m, *Ertuğrul, Tugay*, *Uysal* & *Susanna 2715*, 22.vi.2009 (BC) **[CA3]**	N37° 45.052' E29° 15.995'<br>N41° 5.752' E31° 48.982'<br>N41° 35.356' E32° 41.110'
Centaurea luschaniana Heimerl ex Stapf	C2 Antalya: road Korkuteli-Elmalı, Karamanbeli, 1337 m, *Ertuğrul*, *Tugay*, *Uysal* & *Susanna 2705*, 20.vi.2009 (BC) **[LU]**	N36° 56.681' E30° 09.617'
Centaurea lycaonica Boiss. & Heldr.	C4 Konya: Seydiehir road, 1595 m, *Ertuğrul, Tugay*, *Uysal* & *Susanna 2728*, 25.vi.2009 (BC) **[LY]**	N37° 45.054' E32° 04.529'
Centaurea lycia Boiss.	C2 Antalya: Korkuteli road, 20 km, 768 m, *Ertuğrul*, *Tugay*, *Uysal* & *Susanna 2702*, 20.vi.2009 (BC) **[LYC1]**<br>C2 Antalya: Saklıkent road, Koz Dağı, Taşlı slopes, 1198 m, *Ertuğrul*, *Tugay*, *Uysal* & *Susanna 2700*, 19.vi.2009 (BC) **[LYC2]**	N37° 00.045' E30° 29.409'<br>N36° 53.511' E30° 22.153'
Centaurea wagenitzii Hub.-Mor.	C2 Antalya: Adrasan Bay, south end, 14 m, *Ertuğrul, Tugay*, *Uysal* & *Susanna 2697*, 19.vi.2009 (BC) **[WA1]**<br>C2 Antalya: Adrasan Bay, north end, 20 m, *Ertuğrul, Tugay*, *Uysal* & *Susanna 2698*, 19.vi.2009 (BC) **[WA2]**	N36° 17.817' E30° 28.455'<br>N36° 18.770' E30° 27.812'

Figure S1. Screening for the most likely number of groups (*K*) with non-hierarchical *K*-means clustering (Evanno *et al.*[10]), performed with 100000 independent runs for each value. Structure plots with *K*- from 1 to 10 are shown below.

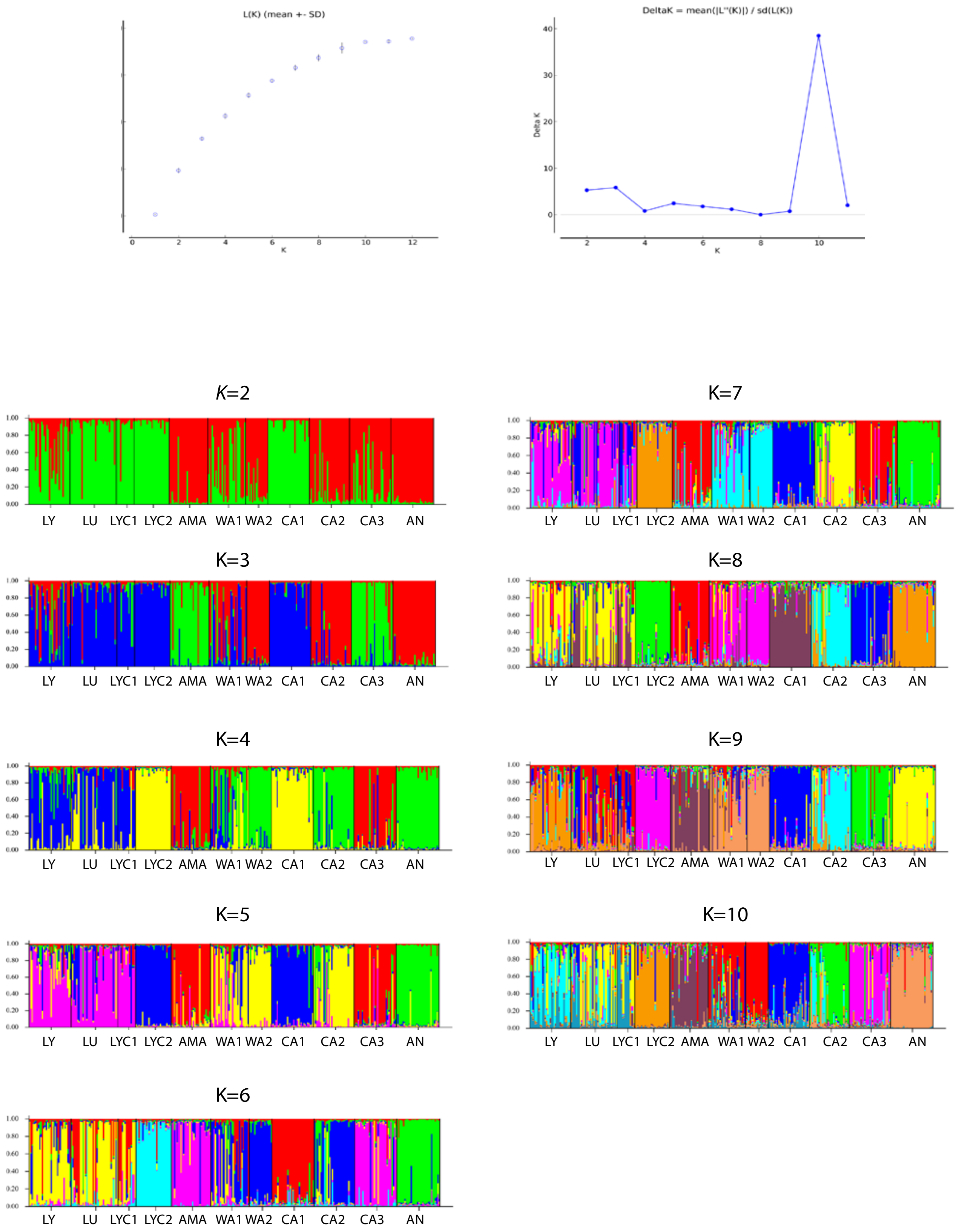

Figure S2. Barrier analysis with 8 barriers, with significance tested by 1000 bootstrap matrices of Nei *et al.*[11] genetic distance (D_A). Numbers indicate bootstrap support (after 8 barriers).

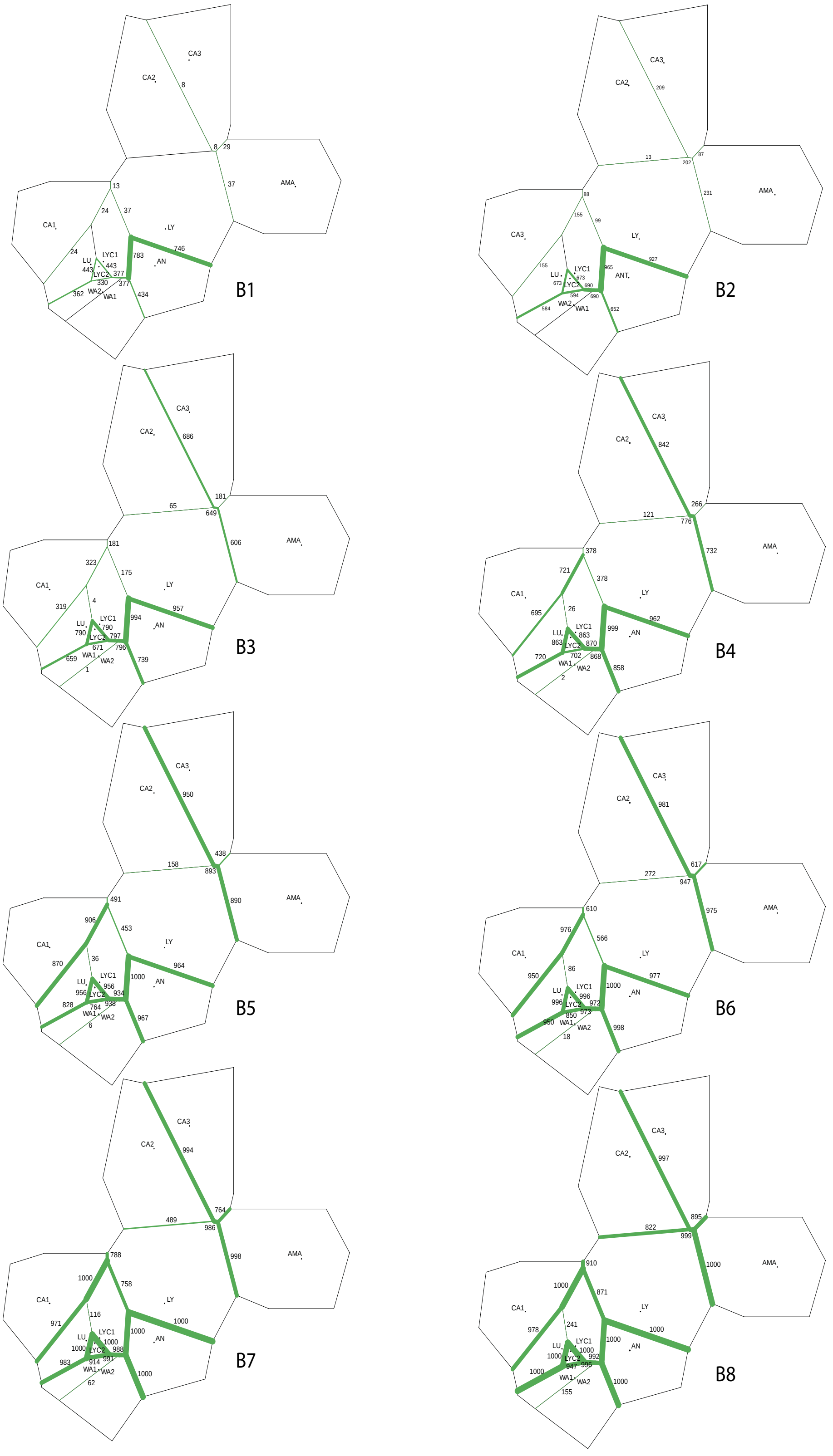

Figure S3. MaxEnt output of the jackknife tests employed to evaluate the relative importance of the nine bioclimatic variables employed in the ecological niche modelling of *Centaurea cadmea*. The variables with highest gain when used in isolation are the most informative. For abbreviations of bioclimatic variables, see Methods.

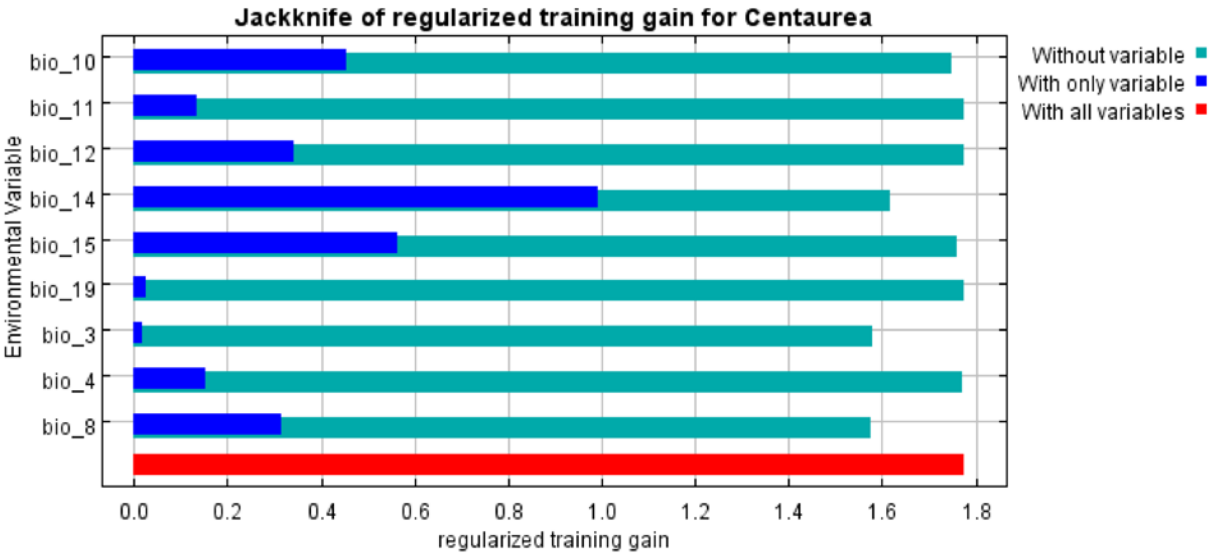

Figure S4. AUC values and success rates (*q*) against several regularization multipliers (0.25, 0.50, 0.75, 1.00, 1.25, 1.50, 1.75, and 2.00). The model with a regularization multiplier of 0.5 is the best model, because it shows the second highest AUC (0.971) while maintaining the highest success rate (0.75).

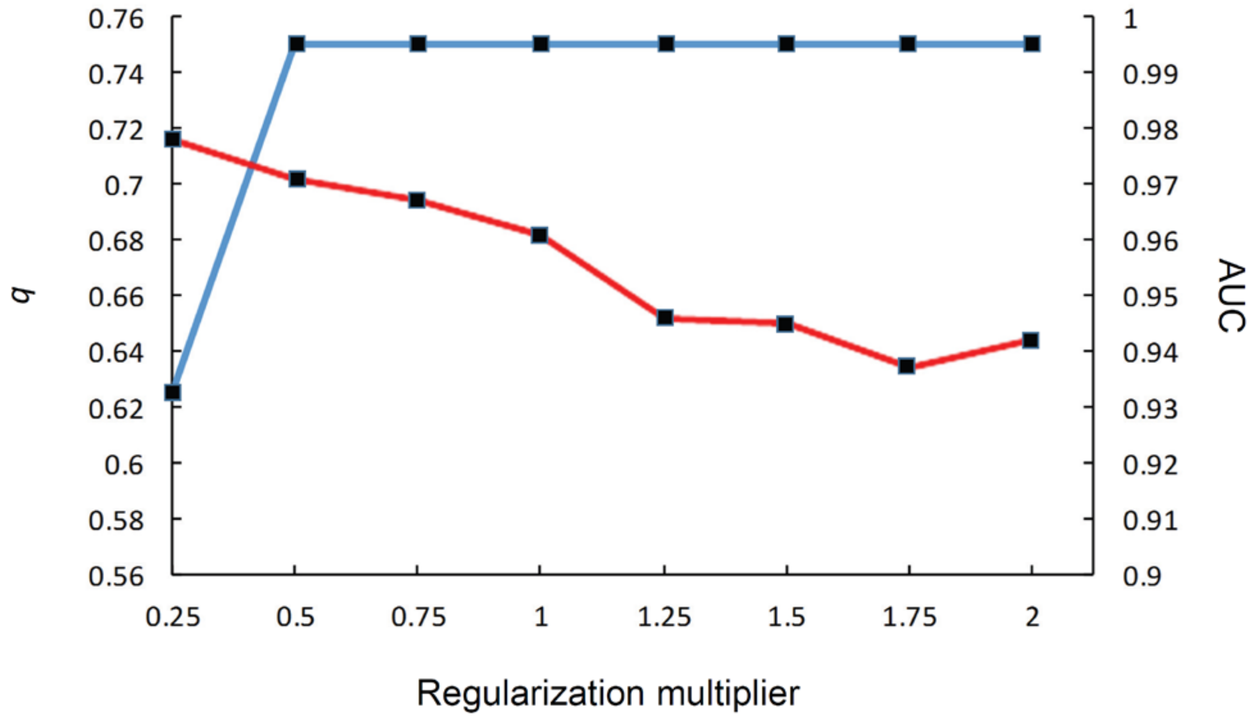

References

1. Guarino, C., Salerno, G. & Cipriani, G. Effects of fragmentation phenomena on the genetic structure and gene flow in *Centaurea cineraria* group (Asteraceae) in the Mediterranean Basin. *Plant Biosyst*. **147**, 996–1005 (2013).
2. Ferriol, M., Merle, H. & Garmendia, A. Microsatellite evidence for low genetic diversity and reproductive isolation in tetraploid *Centaurea seridis* (Asteraceae) coexisting with diploid *Centaurea aspera* and triploid hybrids in contact zones. Bot. J. Linn. Soc. **176**, 82–98 (2014).
3. López-Vinyallonga, S., López-Pujol, J., Constantinidis, T., Susanna A. & Garcia-Jacas, N. Mountains and refuges: genetic structure and evolutionary history in closely related, endemic *Centaurea* in continental Greece. *Mol. Phylogenet. Evol.* **92**, 243–254 (2015).
4. Fréville, H., Justy, F. & Olivieri, I., 2001. Comparative allozyme and microsatellite population structure in a narrow endemic plant species, *Centaurea corymbosa* Pourret (Asteraceae). *Mol. Ecol*. **10**, 879–889.
5. Marrs, R. A., Sforza, R. & Hufbauer, R. A. When invasion increases population genetic structure: a study with *Centaurea diffusa*. *Biol. Invasions* **10**, 561–572 (2008).
6. López-Alvarado, J. Centaurea *L. section* Phrygia *Pers.: Phylogeny and biogeography* (PhD Thesis, Università degli Studi di Sassari, 2012).
7. Mameli, G., Filigheddu, R., Binelli, G. & Meloni, M. The genetic structure of the remnant populations of *Centaurea horrida* in Sardinia and associated islands. *Ann. Bot.* **101**, 633–640 (2008).
8. Marrs, R. A., Sforza, R. & Hufbauer, R. A. Evidence for multiple introductions of *Centaurea stoebe micranthos* (spotted knapweed, Asteraceae) to North America. *Mol. Ecol*. **17**, 4197–4208 (2008).
9. IUCN. *IUCN Red List Categories and Criteria: Version 3.1*. (IUCN Species Survival Commission, 2001).
10. Evanno, G., Regnaut, S. & Goudet, J. Detecting the number of clusters of individuals using the software STRUCTURE: a simulation study. *Mol. Ecol*. **14**, 2611–2620 (2005).
11. Nei, M., Tajima, F., Tateno, Y. Accuracy of estimated phylogenetic trees from molecular data. *J. Mol. Evol*. **19**, 153–170 (1983).
